# Identification of new allosteric sites and modulators of AChE through computational and experimental tools

**DOI:** 10.1080/14756366.2018.1476502

**Published:** 2018-06-06

**Authors:** Carlos Roca, Carlos Requena, Víctor Sebastián-Pérez, Sony Malhotra, Chris Radoux, Concepción Pérez, Ana Martinez, Juan Antonio Páez, Tom L. Blundell, Nuria E. Campillo

**Affiliations:** a Centro de Investigaciones Biológicas (CIB-CSIC), C/Ramiro de Maeztu, Madrid, Spain;; b Department of Biochemistry, University of Cambridge, Cambridge, UK;; c Cambridge Crystallographic Data Centre, Cambridge, UK;; d Instituto de Química Médica (IQM-CSIC), C/Juan de la Cierva, Madrid, Spain

**Keywords:** AChE, allosteric sites, Alzheimer diseases, molecular dynamics, allosteric inhibitors

## Abstract

Allosteric sites on proteins are targeted for designing more selective inhibitors of enzyme activity and to discover new functions. Acetylcholinesterase (AChE), which is most widely known for the hydrolysis of the neurotransmitter acetylcholine, has a peripheral allosteric subsite responsible for amyloidosis in Alzheimer’s disease through interaction with amyloid β-peptide. However, AChE plays other non-hydrolytic functions. Here, we identify and characterise using computational tools two new allosteric sites in AChE, which have allowed us to identify allosteric inhibitors by virtual screening guided by structure-based and fragment hotspot strategies. The identified compounds were also screened for *in vitro* inhibition of AChE and three were observed to be active. Further experimental (kinetic) and computational (molecular dynamics) studies have been performed to verify the allosteric activity. These new compounds may be valuable pharmacological tools in the study of non-cholinergic functions of AChE.

## Introduction

Acetylcholinesterase (AChE) is a well-known enzyme for the hydrolysis of the neurotransmitter acetylcholine (ACh)^1–4^, being the target of the main marketed pharmacological treatment of Alzheimer’s disease (AD). However, during the past decade it has been shown that AChE also plays other non-hydrolytic functions. Several *in vitro* and *in vivo* studies in the central nervous system suggested that developmental regulation of AChE enzyme plays a role in non-cholinergic function as morphometric processes, cell differentiation and synaptogenesis along nervous system^5^. It is well recognised that one of the non-cholinergic actions of AChE is neurite promotion, which is regulated by a dynamic equilibrium of different interactions sites of AChE^6^^,^^7^. Zeev-Ben-Mordehai et al. published that one of the non-classical roles of AChE might be as an adhesion protein involved in synaptic development and maintenance^8^. Thus, pharmacological inhibitors of AChE that block the catalytic activity of the enzyme do not necessarily interfere with other biological activities of the protein. On the contrary, since damaging effects of overexpressed AChE may be related to non-catalytic activities, these drugs may actually aggravate certain conditions by elevating the levels of catalytically inactivated AChE.

To date it is well known that the active site of AChE is subdivided into several subsites: catalytic triad (CAS, Ser-Glu-His) at the base of the gorge, anionic subsite (AS), acyl-binding pocket, and peripheral anionic subsite (PAS) at the mouth of the site, being PAS an allosteric site^9^. Modulators binding PAS limit the catalytic efficiency by both ways, combining steric and electrostatic blockage of ligand trafficking through the gorge and changing the active site conformation. Several evidences suggest that the PAS, besides its role in allosteric regulation of AChE-catalysed hydrolysis, also mediates heterologous protein associations that contribute to cell recognition and adhesion processes during synaptogenesis, and to the nucleation of amyloid peptides during the onset of AD in humans and mammalian model systems^10^.

Recently, Marcelo et al. suggested another possible binding site, called site B, located outside the catalytic gorge. They showed that rosmarinic acid was able to bind this site; however, its allosteric functioning is not clear^11^.

The “*exit doors*” are another very interesting structural motif in the hydrolysis mechanism of AChE. These regions are alternative routes to the gorge for product clearance, contributing to the high catalytic activity of the AChE. Computational studies together with X-ray crystallography suggest three possible regions of AChE implicated in the removal of cleavage products of the hydrolysis of ACh, known as *back door* (including Trp86, Gly448, Tyr449, and Ile451 (*h*AChE residue numbering)). *Side door* (including Asp74, Thr75, Leu76, Thr83, Glu84, and Asn87) and *acyl loop door* (including Trp236, Arg247, and Phe297)^12–15^.

There are many challenges that need to be addressed regarding the non-classical functions of AChE, such as to identify the allosteric sites, the amino-acid residues that mediate non-classical activities and the identification of allosteric inhibitors among others^16^. Taking into account the relevance of this target in neurodegenerative diseases we consider that it is of great importance to identify allosteric sites as starting point to develop efficient modulators of AChE and the corresponding non-cholinergic functions.

The main goal of this work is the identification and description of new allosteric binding sites on AChE and the discovery of new allosteric inhibitors since allosterism represents one of the most common and powerful means to regulate protein function. For this purpose, we performed a search for druggable sites on the enzyme using computational approaches with the aim of identifying putative allosteric sites. Moreover, for each of these we have defined the interacting key residues by means of virtual screening of our Medicinal and Biological Chemistry (MBC) library^17^, and a plausible mechanism of action is proposed.

## Methods

### Computational studies

#### Pocket search

In order to identify different cavities on the AChE enzyme, the Fpocket software^18^, a highly scalable and open source pocket-detection software package, was used. Fpocket is freely available for download at http://www.sourceforge.net/projects/fpocket. Fpocket extracts the information from rigid structure and only it is based on geometric parameters. Fpocket is based on the concept of a α-sphere, which is defined as a sphere that contacts four atoms on its boundary and has no internal atom. For a protein, very small spheres are located inside the protein, large spheres at the exterior, and clefts and cavities correspond to spheres of intermediate radii^19^.

The use of Fpocket software involves three major steps. During the first step, the whole ensemble of α-spheres is determined from the protein structure and a pre-filtered collection of spheres is returned. The second step consists of identifying clusters of spheres close together, identifying pockets, and removing no interesting clusters. The final step is the atom properties calculation from each pocket, in order to score and rank the identified pockets.

Fourteen hAChE structures (PDB IDs: 1B41, 1F8U, 2X8B, 3LII, 4BDT, 4EY4, 4EY5, 4EY6, 4EY7, 4EY8, 4M0E, 4M0F, 4PQE, 5FPQ)^20–27^ were downloaded from the Protein Data Bank (www.pdb.org) and subjected to pocket search using Fpocket. The PDB structures were prepared using the Maestro^28^ Protein Preparation Wizard^29^ for removing the water molecules, ligands, and metal ions. Upon Fpocket search, the 14 structures with embedded centres of pocket α-spheres were analysed by visual inspection to identify conserved pockets.

#### Hotspot analysis

The Fragment Hotspot maps software^30^ identifies the location and quality of binding sites on the protein by first calculating atomic hotspots and then producing Fragment Hotspot maps with simple molecular probes. These maps specifically highlight fragment-binding sites and their corresponding pharmacophores. H-Bond acceptor, donor, and apolar/aromatic interactions, reported by this software, can assist medicinal chemists search for interesting interactions in order to bind or improve the binding affinities for different ligands, and suggest modifications to the molecules. For the molecular modeller, the maps complement existing virtual screening methods because they can be visually inspected to generate docking constraints or structure-based pharmacophores. With the most important interactions highlighted, existing pharmacophore methods can be used to screen for molecules capable of making these essential interactions. The maps can also be used to generate constraints for docking and hence steer the docking towards occupying the hotspot and ensuring that the important potential interactions are satisfied.

Structure preparation of hAChE in its *apo* state (PDB ID: 4EY4) for the hotspot calculation was performed using Protein Preparation Wizard^29^, implemented in Maestro^28^. Ligands and water molecules were removed, hydrogen atoms were added and protein residues were ionised at pH = 7. After the target preparation, Fragment Hotspot maps were calculated using the in-house script developed by Chris Radoux in Cambridge. The Hotspot Maps^30^ were visualised using PyMol^31^ software in order to identify the residues which could be involved in the interaction between ligand and protein.

#### Ligand preparation

The preparation of the library and the 2D-to-3D conversion was performed using the LigPrep^32^ tool, a module of the Schrödinger software package. LigPrep allows different preparation steps of molecules such as the addition of hydrogen atoms, neutralisation of charged groups, generation of ionisation states, low-energy ring conformations, possible tautomers, followed by energy minimisation using the OPLS-2005 force field^33^^,^^34^. In order to carry out our studies, the compounds were prepared at physiological pH conditions, all of the compounds were desalted and finally the compounds were minimised as default. A total of 2499 protonation and tautomeric states were generated from 1830 compounds of MBC library using LigPrep.

#### Virtual screening

Virtual Screening of the MBC library^17^ was carried out using the Glide software^35^, with the Extra Precision (XP) Glide Mode, for the *site 2* and *site 3* of the 4EY4 structure which was previously prepared using Protein Preparation Wizard tool. This structure was selected because of its being the only *apo* form amongst the serie of AChE crystallographic structures^22^; it also has a very good resolution and excellent validation values, such as few Ramachandran outliers. The small-molecule-bound docked poses were further filtered using Maestro Pose Filter, selecting only the compounds that interact through an H-bond with the residues that are known to be critical for the ligand-target affinity in the Hotspots Maps. For *site* 2, the grid was centred on the *site 2*, ensuring that the entire cavity was included inside the box, and the molecules were ranked based on the Glide XP score. The scores for the molecules were in the range from −8.9 to 4.4 kcal/mol while rosmarinic acid was ranked with a score of −8.5 kcal/mol. His405, Glu414, and Trp532 were selected as key residues and applied as interaction filter.

For *site 3*, a Glide XP docking was performed ensuring the entire cavity was included in the grid. The docking score for the molecules ranges from −7.7 to 1.8 kcal/mol. Glu81, Glu452, and Arg463 were selected as key residues for the interaction filtering. Ligands that are able to interact with these residues were ranked by their XP Glide Score, and visual inspection of the fitting between the Hotspot Maps and the best ranked virtual screening results was used to select molecules for further studies.

#### Molecular dynamics

Molecular dynamics (MD) was performed on an Asus 1151 h170 LVX-GTX-980Ti workstation, with an Intel Core i7–6500 K Processor (12 M Cache, 3.40 GHz) and 16 GB DDR4 2133 MHz RAM. The workstation has Nvidia GeForce GTX 980Ti available for GPU computations. MD studies were performed using AMBER14^36^ with the ff14SB^37^ to assess the stability of the compounds and to look for the ligand’s inhibitory mechanism. Additionally, a MD trajectory for the *apo*-target was developed, in order to observe the behaviour of the *apo*-AChE enzyme and to compare it with the ligand-target trajectories. To calculate the ligands parameters for the MD simulation, RESP charges were calculated using Gaussian09^38^, optimising the geometry for both compounds using the method HF6–31++(d,p). Once the optimisation was completed, ligands were parametrised using Antechamber module^39^. Systems were solvated using TIP3P model^40^ for water molecules, with a cubic box, equilibrating the system charge by adding Na^+^ ions. Solvated systems were first minimised for 8000 steps with the initial 4500 steps using the steepest descent algorithm. The final 3500 steps used the conjugate gradient energy minimisation with constraints applied to the protein residues as mentioned above for *sites 2 and 3*. This was followed by two minimisation stages of 8000 steps each, with the last 3500 using the conjugate gradient decreasing the restrains to the system. The system was equilibrated to 300 K and 1 atm, using a step protocol, applying energetic restraints of 15 kcal mol^−1 ^Å^−1^ from the initial step and gradually decreasing them until its disappearing. Trajectories of 25 ns were obtained in isothermal–isobaric ensembles. All bonds involving hydrogen atoms were constrained with the SHAKE algorithm^41^. A cut-off of 10 Å was used for the Lennard–Jones interaction and the short-range electrostatic interactions. Berendsen barostat^42^ and Langevin thermostat were used to regulate the system pressure and temperature, respectively. Trajectories of 25 ns were computed, analysed using the Cpptraj^43^ module and VMD^44^ for visual inspection. Xmgrace software^45^ was used to obtain the graphics of root-mean-square deviation (RMSD) and root-mean-square fluctuation (RMSF) of the MD simulations.

One of each five frames of the trajectories were saved into a new PDB format trajectory and were taken to further analysis using TRAnsient Pockets in Proteins (TRAPP) software^46^, which allows the simulation, analysis, and visualisation of protein cavity dynamics for detection of transient sub-pockets using protein motion trajectories or ensembles of protein structures obtained either from experiments or from simulations. The catalytic pocket was also analysed using this software.

### Biological studies

#### *In vitro* cholinesterase inhibition assays

The method was adapted from Ellman et al.^47^ The assay solution consisted of 0.1 M phosphate buffer pH 8, 400 mM 5,5′-dithiobis(2-nitrobenzoic acid) (DTNB, Ellman’s reagent), 0.05 unit/ml AChE (Sigma Chemical Co., Madrid, Spain, Cholinesterase, acetyl human recombinant), and 800 mM acetylthiocholine iodide as the substrate of the enzymatic reaction. The compounds tested were added to the assay solution and the absorbance changes at 412 nm were recorded for 5 min with a UV/Vis Microplate and cuvette Spectrophotometer, Thermo Electron Type, Multiskan Spectrum. The reaction rates were compared, and the per cent inhibition due to the presence of test compounds was calculated. The IC_50_ is defined as the concentration of each compound that reduces the enzymatic activity 50% with respect to that without inhibitors. All the experiments were performed in triplicate.

#### Kinetic study of AChE inhibition

To investigate the mechanism of action of the compounds on AChE, a kinetic analysis was performed. The experiments were carried out using combinations of four substrate concentrations, and three inhibitor concentrations. Double-reciprocal Lineweaver–Burk plotting of the data obtained, in which each point is mean of three different experiments, were analysed.

Competitive inhibitors have the same *y*-intercept as uninhibited enzyme (since *V*_max_ is unaffected by competitive inhibitors the inverse of *V*_max_ also does not change) but there are different slopes and *x*-intercepts. Non-competitive inhibition produces plots with the same *x*-intercept as uninhibited enzyme (*Km* is unaffected) but different slopes and *y*-intercepts. Non-competitive inhibition causes different intercepts on both the *y*- and *x*-axes but the same slope. Mixed inhibitors cause intersects above or below the *x*-axis.

## Results and discussion

### Druggable site

The determination of druggable cavities in therapeutic targets is essential for structure-based drug design in order to identify binding pockets or allosteric sites and to design small-molecule ligands that bind to these with therapeutic effects. To address this goal, several computational approaches have been developed based mainly on evolutionary or structure-based algorithms^48–51^.

In this work, we have used a combination of methods to get a consensus prediction of protein druggable sites. We have used the free geometry-based algorithm Fpocket^18^^,^^19^ (http://fpocket.sourceforge.net) together with the prediction of Fragment Hotspot maps^30^ to study the AChE surface with the aim to identify allosteric sites and its key residues.

The first step of this consensus protocol was performed using the Fpocket software. As Fpocket is a geometry-based pocket-detection algorithm, we performed the study using a representative set of hAChE, in both *apo* and different complexed forms (Table 1). Fourteen different human structures were available at the moment of the study in the Protein Data Bank (PDB) (see Material and Methods section). The software detected almost 30 cavities, with some of these appearing in all AChE structures analysed, and others, which are not conserved, in fewer crystal structures.

**Table 1.** Druggable binding sites.

PDB code	LIGAND	*SITE 1*	*SITE 2*	*SITE 3*		*SITE 4*
1B41	Fasciculin-II	1	4, 9	2	3	
1F8U	Fasciculin-II	1	4, 7	3	2	
2X8B	Fasciculin-II	1	9, 11	6	2	
3LII	–	1	8, 17	2	7	
4BDT	Fasciculin-II	1	4, 10	2	5	
4EY4	–	2	5, 7	1	4	
4EY5	Huperzine	1	4, 6	2	3	
4EY6	Galantamine	1	2, 4	3	5	
4EY7	Donepezil	1	4, 6	2	3	
4EY8	Fasciculin-II	1	6, 10	2	3	
4M0E	Dihydrotanshinone-I	1	4, 7	2	6	
4M0F	Territrem B	1	4, 5	2	3	
4PQE	–	1	2, 3	5, 13	4	
5FPQ	Sarin	1	3, 5	2	4	

The numbers refer to the score given by the program Fpocket to the site in each structure (the lower score is related to better binding sites). Two or more numbers indicate that this pocket was found as two different cavities on the structure.

**Figure 1.**
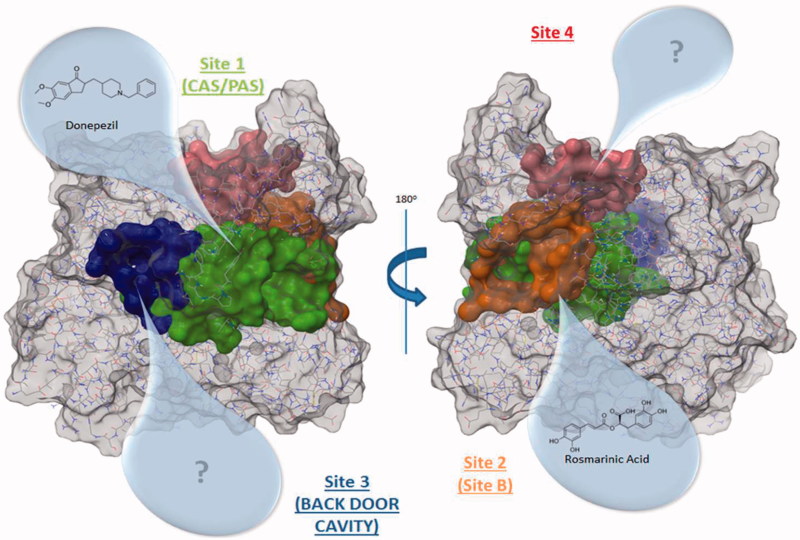
AChE structure highlighting the best cavities found by Fpocket.

Once all cavities were analysed and clustered, taking into account the frequency that they appear together, and the druggability prediction score given by Fpocket, only four highly reproducible cavities can be considered as putative druggable binding sites (Figure 1). We observed that the four sites appear in all of the different structures, although the position of each site varies among the structures and, in the case of *site 2*, Fpocket detected it as two different cavities (Table 1).

**Figure 2.**
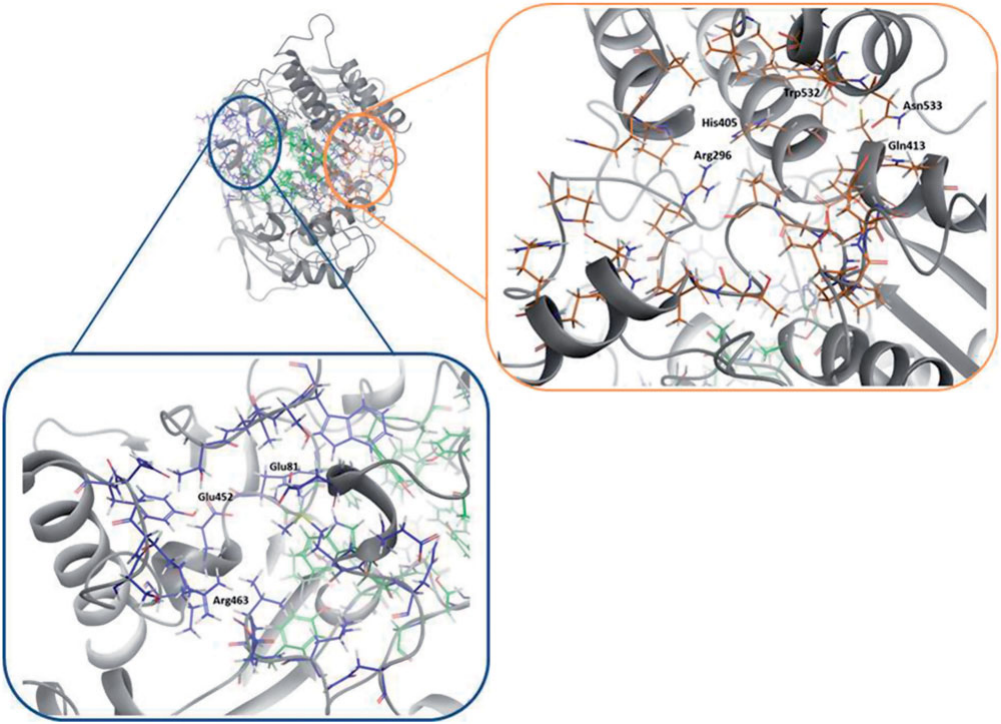
Amino acids involved in *site 2* and *site 3* found in hAChE (PDB ID: 4EY4). *Site 2* residues are shown in orange and *site 3* residues are shown in blue.

The best ranked site for all structures is the *site 1* (except for 4EY4, for which is the second-best cavity) that corresponds to the well-known binding site CAS/PAS. The *site 2* is ranked at the top five positions in 11 out of the 14 structures analysed. Inspection of the residues of this site (Table 2) allowed us to identify Arg296 and Glu369 as possible key binding residues of the rosmarinic acid as was previously described by Marcelo et al.^11^ Some residues are also shared with the *acyl loop door*^14^ such as Trp236, Arg247, and Phe297 (Figure 2).

**Table 2.** Residues of allosteric sites, *site 2* and *site 3.*

Allosteric site	Residues
*Site 2*	Pro232, Asn233, Gly234, Pro235, Trp236, Thr238, Val239, Gly240, Glu243, Arg246, Arg247, Leu289, Por290, Gln291, Ser293, Arg296, Phe297, Val300, Thr311, Pro312, Glu313, Pro368, Gln369, Val370, Asp404, His405, Cys409, Pro410, Gln413, Trp532, Asn533, Leu536, Pro537, Leu540
*Site 3*	Glu81, Gly82, Glu84, Met85, Asn87, Asn89, Leu130, Asp131, Val132, Thr436, Leu437, Ser438, Trp439, Tyr449, Glu452, Ile457, Ser462, Arg463, Asn464 y Tyr465

The *site 3*, in 13 out of the 14 structures analysed, is ranked in the top five positions. We identified residues Val132, Tyr449, and Glu452 as residues (Table 2) that take part of the so-called *back door*, described as a dimple on the surface of the protein^52^.

The remaining *site 4* corresponds to a cavity formed mostly by polar residues such as Asp333, Glu334, His381, Glu396, and Asp400. Since no molecular or biological function can be attributed to these residues, we did not pursue the study of this cavity. However, the possibility of using *site 4* for the design of new allosteric modulators of AChE remains open for future work. The known *side door*^53^^,^^54^ was also detected by Fpocket, although this cavity was not found in most of the structures (see Supporting Information).

### Volume characterisation

**Figure 3.**
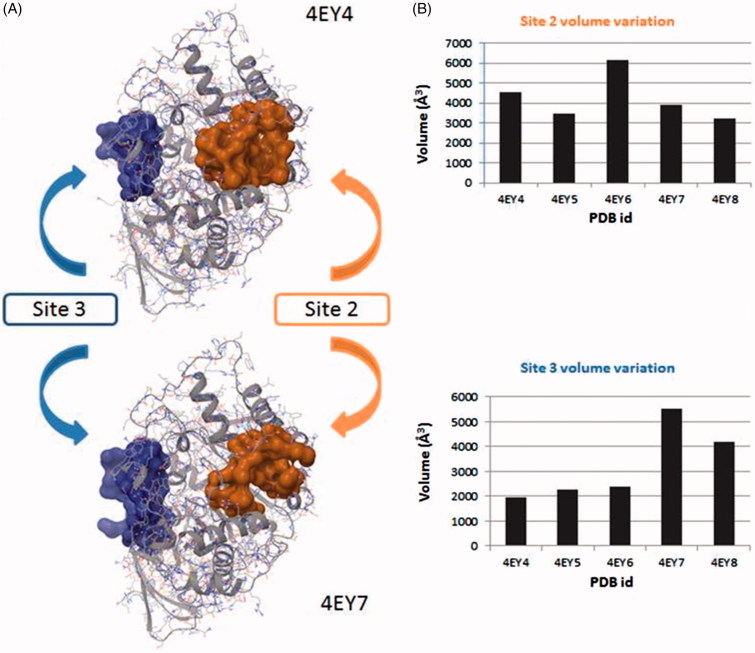
(A) Surface representation of pockets that belong to *site 2* (orange) and *site 3* (blue). Differences between the volume in the absence and presence in the CAS/PAS cavity of inhibitor are highlighted (4EY4 is AChE in the apo state, 4EY7 is AChE crystallised with donepezil). (B) Plots of volume measured in some structures crystallised in the same conditions for *site 2* (orange) and *site 3* (blue).

Further we noticed that there was a fluctuation in the volume of the *site 2* and *site 3* when a ligand is bound in catalytic gorge. To analyse these fluctuations we use Fpocket to compare a series of structures crystallised in the same conditions (4EY4, 4EY5, 4EY6, 4EY7, 4EY8). We observed that the *site 2* decreases its volume when a ligand is bound to the catalytic gorge except to complex 4EY6 (Figure 3). These changes in the volume present a logical issue, because *site 2* and CAS have some common residues, so ligands in the active gorge can interact and displace these residues as a consequence and hence modify the contiguous cavity. Similar changes might affect the active gorge when a ligand binds the *site 2*, explaining a possible allosteric mechanism. Moreover, it has been reported that the structural perturbations of the acyl loop occur when AChE is inhibited by a covalent inhibitor, presenting a narrowing of the gorge and a displacement of the Arg296 into the active site, which could present a steric barrier to the entry of oximes or affect oxime binding for AChE reactivation^55^. In our case, the hypothesis that a ligand binds to *site 2* and displaces the acyl loop, thus inhibiting AChE activity, will be further studied.

We next studied the influence of the ligand in complexes 4EY7 (with donepezil) and 4EY8 (with fasciculin-2) on the volume of the *site 3*. Interestingly, we found that in both cases the *site 3* rises its volume approximately by two-fold as compared to the *apo* structure. This could be explained by the opening of the *back door* in both complexes, while the others remain closed.

These results, indicative of a volume fluctuation of allosteric sites, get with Fpocket are preliminary but suggesting an interrelation between catalytic site and allosteric ones.

### Key residues

In order to confirm the *druggability* of the allosteric sites and to identify its key residues, we next performed a theoretical study of potential fragment-binding sites using Fragment Hotspot maps^30^ for each site detected by Fpocket. These hotspot maps provide visual guides of the fragment-binding sites and their corresponding pharmacophores. This method reports H-Bond acceptor, donor, and non-polar/aromatic interactions, helping in the search for interesting interactions in order to identify or design efficient inhibitors.

**Figure 4.**
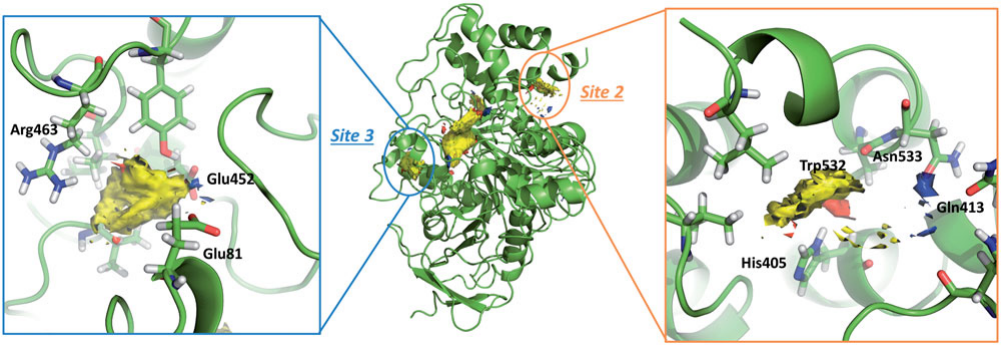
Representation of the calculated hotspots using Fragment Hotspot maps software^30^. Yellow area refers to non-polar area where the ligand should make hydrophobic interactions. Blue dots represent the area where the ligand should make a donor H-bond, and red dots where acceptor H-bond should be formed.

Figure 4 shows the Fragment Hotspot maps of the AChE *apo* structure (PDB ID 4EY4). The analysis of the hotspot was focussed on *sites 2* and *3*. We found that key residues Gln413 and Trp532 in *site 2* can act as acceptors and His405 as H-Bond donor. These interactions represent the theoretical minimum binding features that will allow a fragment to bind this cavity.

Fragment Hotspot maps for the *site 3* were also analysed, demonstrating that the key residues are Glu81 and Glu452 as acceptors and Arg463 as a H-Bond donor.

In summary, considering all above results we can conclude that druggable sites (*sites 2* and *3*) could be possible allosteric sites and targeting them might lead to the modification of AChE activity.

### Virtual screening

With the goal of searching for allosteric modulators of the *sites 2* and *3*, we performed a virtual screening using our in-house MBC chemical library^17^. To validate the computational screening, docking of donepezil and rosmarinic acid for both sites (CAS/PAS and *site 2*) was also performed using Glide software (see Supporting Information, Figures S1 and S2). Upon validation, a virtual screening was performed using Glide with XP for both sites.

#### Site 2 virtual screening

Eleven compounds were chosen for enzymatic assay. Two of these compounds were selected based on their docking score. The rest of them were selected by an interaction-filter based on the Fragment Hotspot maps that were previously calculated. In this way, a filter of the capability of the ligands to interact through H-bond with Gln413, Trp532, and His405 was set up and the results were visually inspected. Along with the 11 compounds selected, rosmarinic acid was also sent for biological evaluation on AChE as a control^11^ (Table 3).

**Table 3.** Experimental inhibition values of the virtual screening compounds to bind *site 2.*

Compounds	Structure	IC_50_ (μM)^a^	Interactions (H-bonds)
VSP2.47		>10 (35%)	Asn233, His405
DA003		>10 (40%)	His405, Trp532
SC274		>10 (33%)	Asn233, Asn533, Trp532
AEL039		>10 (45%)	Asn233, His405, Trp532
SC653		>10 (26%)	Trp532, Asn233
JHD1.21		>10 (33%)	Asn533, His405
MR3.61		>10 (37%)	Asn233, Asn533, His405
AEL011		>10 (44%)	Asn533, His405
SC251		2.76 ± 0.25^b^	Gln413, Asn533, His405
VP2.42		>10 (39%)	Trp532
SC008		>10 (38%)	His405, Trp532
Rosmarinic acid		>10 (26%)	Asn533, Gln413, Thr238, Pro368, Arg296

Key interactions of the compounds with AChE are display.

^a^% of inhibition at 10 μM is indicated into parentheses.

^b^IC_50_ curve of compound SC251 (see supporting information, Figure S3).

All the compounds tested showed a percentage of AChE inhibition at 10 μM greater than rosmarinic acid (the control compound in the assay), consistent with the proposed common binding site in the enzyme. Very interesting is the case of pteridine derivative SC251 with an IC_50_ value of 2.7 μM.

**Figure 5.**
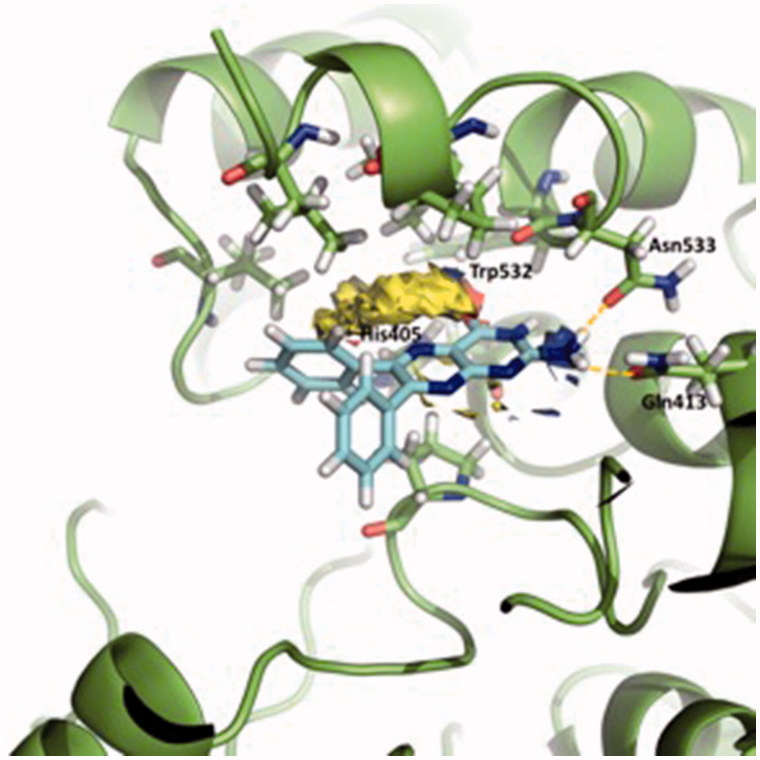
Superposition of the proposed pose for SC251 at *Site 2* and the hotspot calculated with Fragment Hotspot maps software.

Figure 5 displays a three-dimensional view of the predicted binding pose of SC251 at *site 2*. SC251 interacts by a donor hydrogen bond with Gln413, an acceptor H-bond with His405 and a donor H-bond with Asn533, fitting properly with the Fragment Hotspot maps results.

**Figure. 6.**
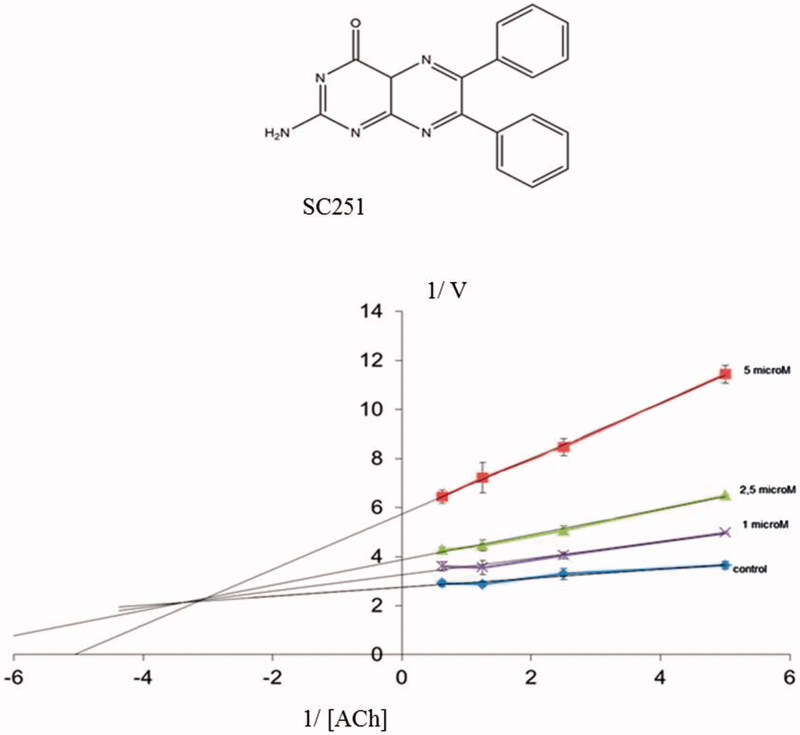
Lineweaver–Burk plots representing the reciprocal of initial enzyme velocity versus the reciprocal of ACh concentration in the absence and presence of different concentrations of SC251 (1–5 μM). Each point is the mean of three different experiments.

To confirm a possible allosteric mechanism of inhibition, we perform competitive studies of SC251 with the natural substrate, acetylcholine (ACh) (Figure 6). A non-competitive-mixed mechanism was observed suggesting that SC251 should bind to an allosteric site, in addition to the catalytic site.

### MD

With the aim of explaining a plausible mechanism of action for the allosteric inhibitor SC251, MD studies for AChE in the *apo* state and AChE-SC251 complex were carried out. MD studies provide a powerful method to follow intimate details of the conformational events in different biological systems. In this study, flexibility changes or behaviour modifications in the important sites of AChE upon inhibitor binding, at the *sites 2* and *3*, and the *side door* cavity were further analysed.

To explore the dynamic stability of the trajectories, RMSD values of the protein backbone based on the starting structure along the simulation time were calculated and plotted (Figure S4). After the initial adjustment, the longer term RMSD values are stable, ranging from 1.0 to 1.9 Å during the entire simulation time.

Along the trajectory, the ligand SC251 fluctuates around the predicted docking poses (Figure S5). Accordingly, during the simulation, the ligand maintains some of the strong interaction as the H-bond donor with the Asn533 (during the 85% of the simulation) and the H-bond acceptor with the His405 (during the 50% of the simulation). However, the H-bond donor with Gln413 is lost in the majority of the simulation, and appears only 2% of the simulation time as it can be observed in Figure S6 (in order to clarify the final bind mode of the complex, a picture is shown in Figure S7).

The RMSF of the *apo* and bound state were compared in order to see any differences in the fluctuations of the residues (Figure S8). The most interesting change corresponds to the residues 70–78, that belong to the omega loop, that are also implicated in the *side door*. We can identify rigidity of these residues when SC251 binds the *site 2*, as demonstrated by the decrease in fluctuations from approximately 3 Å to barely 1 Å, which might suggest the impossibility of opening the *side door* while the ligand remains bound to the *site 2.*

**Figure 7.**
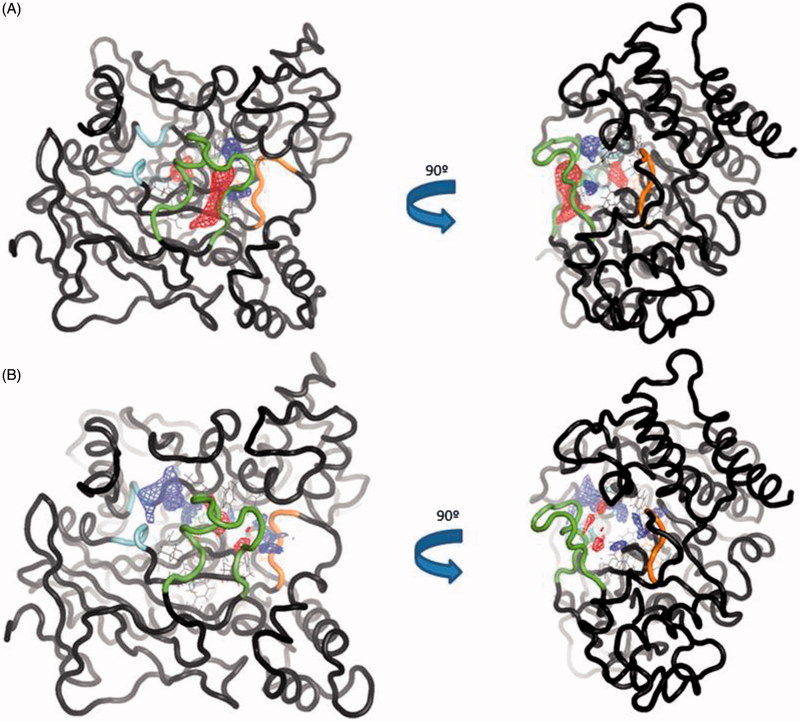
(A) TRAPP analysis for the *apo* AChE trajectory. (B) TRAPP analysis for the AChE-SC251 trajectory. Blue areas represent disappearing areas at the 50% of the snapshots, red areas represent appearing areas at the 50% of the snapshots. Green loop corresponds to the *side door*, orange loop to the acyl-loop and the blue residues to the *back door*.

To further analyse the conformational changes between the trajectories, we used TRAPP software^46^. TRAPP is a tool that allows the analysis of the evolution of the spatial and physicochemical properties of a specified pocket in a protein during a MD simulation. We selected one out of five frames of the simulation as an input for TRAPP. We first analysed the CAS/PAS cavity of the *apo* AChE trajectory, which reveals that our molecular dynamic study also shows the presence of the *side* and *back door*s in at least the 50% of the trajectory, as other previous studies have already published^56^ (Figure 7(A)). The *acyl loop door* is less flexible therefore the gate opening is more difficult to occur, as other studies have also previously reported^56^, thus we only can see its opening in 25% of the total snapshots of the simulation.

Analysing the CAS/PAS cavity of the complex AChE-SC251 trajectory, some interesting differences with the *apo* form are highlighted. As we commented above, while the omega loop of the *apo* state of AChE is very flexible and allows the opening of the *side door*, in the AChE-SC251 trajectory this loop is much more rigid and as a result, the *side door* opens only in <50% of the snapshots of the trajectory. A red area appears in the *back door* region of the *apo* AChE trajectory, indicating a transient pocket when the *back door* is opening (Figure 7). However, for the AChE-SC251 trajectory we can see instead a big disappearing area (in blue, Figure 7), suggesting the impossibility for this door to open. Another difference is also noticed near the acyl loop, while the loop remains rigid in the *apo* trajectory, for the AChE-SC251 trajectory a disappearing area is situated near it, suggesting a slight displacement of these residues into the CAS/PAS cavity.

In general terms, we observe a stiffness of the doors. All of these data are in agreement with the allosteric theory that proposes that the binding of a ligand can reduce the entropy of the system in such a way that the conformation of the protein is fixed, restricting its movement and thus modifying the target behaviour^57^.

#### Site 3 virtual screening

For the *site 3*, no ligands can be used as a control for docking calculations since no previous studies have been carried out. Thus, the same docking conditions as for the virtual screening in the *site 2* were used, following Fragment Hotspot maps leads.

In this case, 14 compounds were selected for biological evaluation using the hotspots calculated as a filter for the screening. Four different filters were set, taking into account the ability of the ligands to make an H-bond interaction with Arg463, Glu452, and Glu81.

**Figure 8.**
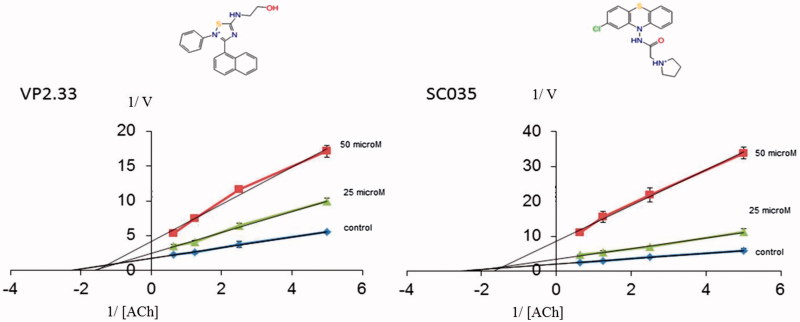
Lineweaver–Burk plots representing the reciprocal of initial enzyme velocity versus the reciprocal of ACh concentration in the absence and presence of different concentrations of VP2.33 and SC035. Each point is the mean of three different experiments.

As given in Table 4, two compounds, VP2.33 and SC035, showed an IC_50_ of ∼50 μM. A competition study for both was performed showing a non-competitive inhibition. At larger concentrations of substrate, the inhibition percentage slightly decreases, as shown in graphic of Figure 8.

**Table 4.** Experimental inhibition value of the identified compounds to bind *site 3* and binding interactions with AChE.

Compound	Structure	IC_50_ (μM)^a^	Interactions (H-bonds)
SC867		>50	Arg463, Tyr465
AC088		>50	Glu81
SC507		>50 (24%)	Glu452
SC872		>50	Glu452, Arg463
SC003		>50	Glu452, Arg463
SC319		>50	Glu81, Glu452, Arg463
AEL040		>50	Glu81, Ser438
AC051		>50	Glu81
SC484		>50	Glu81
VP2.33		49.6 ± 1.5^b^	Glu81
VNG1.9		>50	Glu81, Asn464
SC035		42.1 ± 4.3^b^	Arg463, Tyr465
SC045		>50	Glu452, Arg463, Tyr465
VP1.58		>50	Glu81, Glu452, Thr436

^a^% of inhibition at 50 μM is indicated into parentheses.

^b^IC_50_ curve of compounds VP2.33 and SC035 (see supporting information, Figures S9 and S10).

Clearly, both compounds bind to AChE at an allosteric site. To further validate the allosteric behaviour of these compounds we measured their inhibitory capacity in the presence of the pure competitive inhibitor JTE-907^58^. If our hypothesis is right, the binding of VP2.33 and SC035 to the allosteric *site 3* in presence of JTE-907 should show a cooperative behaviour in terms of inhibition of the AChE.

Table 5 shows the inhibitory value of each compound and their sum. In the case of VP2.33 the data indicate an effect of cooperative activities. These data support a model where both compounds collaboratively participate in the inhibition of AChE by targeting different sites in the enzyme.

**Table 5.** Results of the inhibition of AChE with JTE-907 and VP2.33.

	VP2.33 50 μM	VP2.33 25 μM
	(57.71 ± 1.38%)	(27.28 ± 3.51%)
JTE907 20 μM (65.10 ± 2.37%)	90.06 ± 1.05	80.76 ± 1.15
	ΔI_JTE907_ = 24.96	ΔI_JTE907_ = 15.66
	ΔI_VP2.33_ = 32.35	ΔI_VP2.33_ = 53.48
JTE907 10 μM (56.78 ± 1.35%)	85.42 ± 1.74	68.57 ± 1.91
	ΔI_JTE907_ = 28.74	ΔI_JTE907_ = 11.79
	ΔI_VP2.33_ = 27.71	ΔI_VP2.33_ = 41.29
JTE907 5μM (30.34 ± 2.85%)	74.87 ± 2.01	50.20 ± 2.73
	ΔI_JTE907_ = 44.53	ΔI_JTE907_ = 19.86
	ΔI_VP2.33_ = 17.16	ΔI_VP2.33_ = 22.90

The value in parentheses corresponds to individual inhibition of each compound.

However, when the experiment was performed with SC035, there is no addition of activities (Table S1) and therefore the results are not conclusive for this compound.

**Figure 9.**
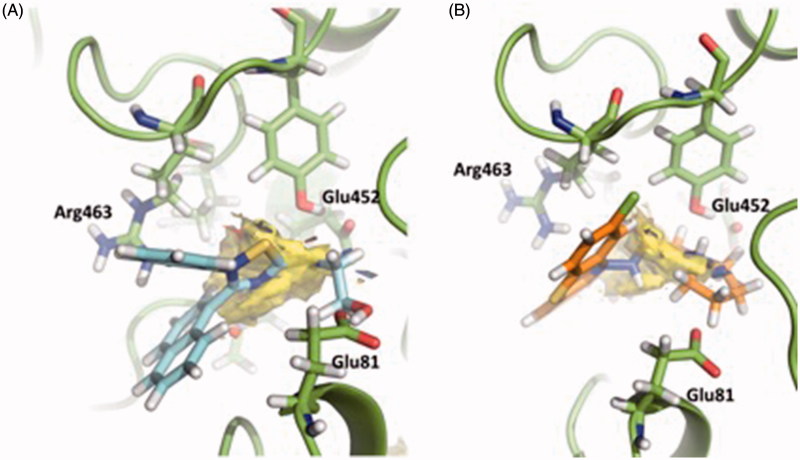
Superimposition of the proposed pose for VP2.33 (blue) (A) and SC035 (orange) (B) at *Site 3* and the hotspot calculated with Fragment Hotspot maps software.

Since the interactions of SC035 and VP2.33 are different (Figure 9), both compounds could be inhibiting the enzymatic activity but with different mechanisms of action, explaining the difference when the sum of activities is measured.

### Molecular dynamics

Further MD studies have been carried out to study the behaviour of the complex AChE-VP2.33 in order to propose a possible mechanism of action of this compound.

The RMSD values of the protein backbone based on the starting structure along the simulation time were calculated and plotted (Figure S11). After the initial adjustment, the longer term RMSD values are stable, ranging from 1.2 to 1.8 Å during the entire simulation process.

Along the simulation of the AChE-VP2.33 complex, we found that the compound pose remains stable along the trajectory (Figure S12), maintaining the H-bond donor interactions with the Glu452 (during the 95% of the simulation) and H-bond donor and acceptor interactions with the Thr436 (during 30% of the simulation) (Figures S13–S14).

The RMSF of the *apo* and bound forms were computed and compared in order to see any differences in the fluctuations of the residues (Figure S15). One of the differences lie on the omega loop residues, in which a decrease in the loop flexibility occurs when VP2.33 binds the *site 3*. Other two regions that also lose flexibility are residues 251–260 and residues 330–347 that comprise loops exposed to the solvent. This rigidity process, when VP2.33 binds AChE, suggests the stabilisation of the complex, reducing its Gibbs free energy and entropy values^59^.

**Figure 10.**
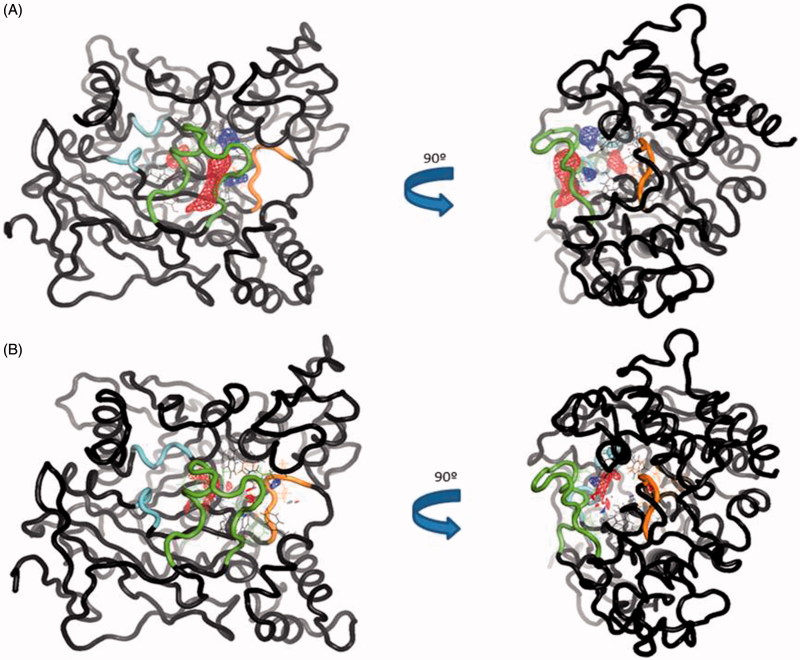
(A) TRAPP analysis for the *apo* AChE trajectory. (B) TRAPP analysis for the AChE-VP2.33 trajectory. Blue areas represent disappearing areas at the 50% of the snapshots, red areas represent appearing areas at the 50% of the snapshots. Green loop corresponds to the *side door*, orange loop to the acyl-loop and the blue residues to the *back door*.

To verify these changes, we studied different areas of AChE along the MD trajectory using TRAPP software. The normal behaviour of the omega loop that corresponds to the opening of the *side door* is modified when the ligand is bound to the target. In the *apo* form a red area appears (new transient pocket) pointing out the opening of the *side door*, meanwhile this new transient pocket does not appear in the complex AChE-VP2.33 (Figure 10). This fact could suggest that compound VP2.33 could be able to modify the behaviour of the omega loop residues, as shown in the RMSF data in Figure S4.

Regarding the *back door*, significant differences are not appreciated in the movement of the residues involved in the opening of this door between the two trajectories. This suggests that the compound VP2.33 might block the clearance of ACh, without modifying the movement of the residues involved in the *back door*.

In summary, we do not find any significative changes in acyl loop neither *back door*. Therefore, we can suggest an innovative allosteric mechanism of VP2.33 due to the prevention of *side door* opening. The residues of this alternative channel present less movement when VP2.33 is bound to *site 3* than the *apo* trajectory correlating with a lower efficiency of the clearance of the degradation products of ACh.

## Conclusions

Our main goal in this work was to extend knowledge of the druggable sites of AChE. Since allosterism represents one of the most common and powerful means to regulate protein function, we aimed to study the AChE surface to identify allosteric sites. A combination of the free geometry-based algorithm Fpocket with the Fragment Hotspot maps have allowed the identification of new allosteric binding sites (*sites 2* and *3*). We carried out a virtual screening study of our in-house library to identify allosteric inhibitors of *sites 2* and *3.* We validated the predicted hits with experimental studies (*in vitro* and kinetic studies). These studies have culminated with the identification of allosteric compounds. SC251 has been identified as an allosteric inhibitor of* site 2* showing a non-competitive-mixed inhibition. Further MD allowed us to propose a possible action mechanism of this compound. In relation to *site 3*, VP2.33 and SC035 have been identified. Both compounds show a non-competitive inhibition. Further experimental studies (cooperative activities) have allowed us to validate VP2.33 as an allosteric inhibitor of *site 3*. These new allosteric modulators are potentially useful pharmacological tools for study of non-hydrolytic functions of cholinergic system.

## Supplementary Material

Supplemental Material
