# Supplementary material for: Identification of new allosteric sites and modulators of AChE through computational and experimental tools
Source: J Enzyme Inhib Med Chem. 2018 Jun 6;33(1):1034–47. doi: 10.1080/14756366.2018.1476502 (PMC6010107; doi:10.1080/14756366.2018.1476502)
Supplement: Supplemental Material [file IENZ_A_1476502_SM3841.pdf]

Supporting Information

Identification of new allosteric sites and modulators of AChE through computational and experimental tools.

Carlos Roca[1&], Carlos Requena[1&], Víctor Sebastián-Pérez[1], Sony Malhotra[2], Chris Radoux[2,4], Concepción Pérez[3], Ana Martinez[1], Juan Antonio Páez[3], Tom L. Blundell[2] and Nuria E. Campillo*[1]

[1] Centro de Investigaciones Biológicas (CIB-CSIC). C/ Ramiro de Maeztu, 9, 28040, Madrid, Spain

[2] Department of Biochemistry (U. Cambridge). 80 Tennis Ct Rd, Cambridge CB2 1GA, UK

[3] Instituto de Química Médica (IQM-CSIC). C/ Juan de la Cierva, 3, 28006, Madrid, Spain

[4] Cambridge Crystallographic Data Centre, 12 Union Road, Cambridge, CB2 1EZ, UK

[§]Theses authors have contributed equally to this work

[*]Corresponding author

Dr. Nuria E. Campillo (nuria.campillo@csic.es)
Centro de Investigaciones Biológicas (CSIC)
Ramiro de Maeztu 9, 28040 Madrid-Spain
Tel. +34 91 838 31 12

Docking protocol validation

Indeed, the best docking solution for donepezil reproduces in a similar way the experimental AChE-donepezil complex (PDB ID: 4EY7) (Figure S1) with very good scoring and energy values. Meanwhile the predicted pose for *site 2* shows energy values and scoring worse than for CAS/PAS site, (-12.1 kcal/mol against -4.3 kcal/mol). Under the same docking conditions, Glide predicted similar binding affinities for rosmarinic acid in both pockets (-10.9 kcal/mol for site CAS/PAS and -8.5 kcal/mol for *site 2*). However, analysing the poses of rosmarinic acid in each site, it is possible to observe that meanwhile Glide predicts solutions in site CAS/PAS quite different from that of donepezil, the solution predicted for *site B* agrees with the hotspots found on this structure, making a donor H-bond with the 4-hydroxy group of the rosmarinic acid and the backbone of the Glu413 residue (Figure S2).

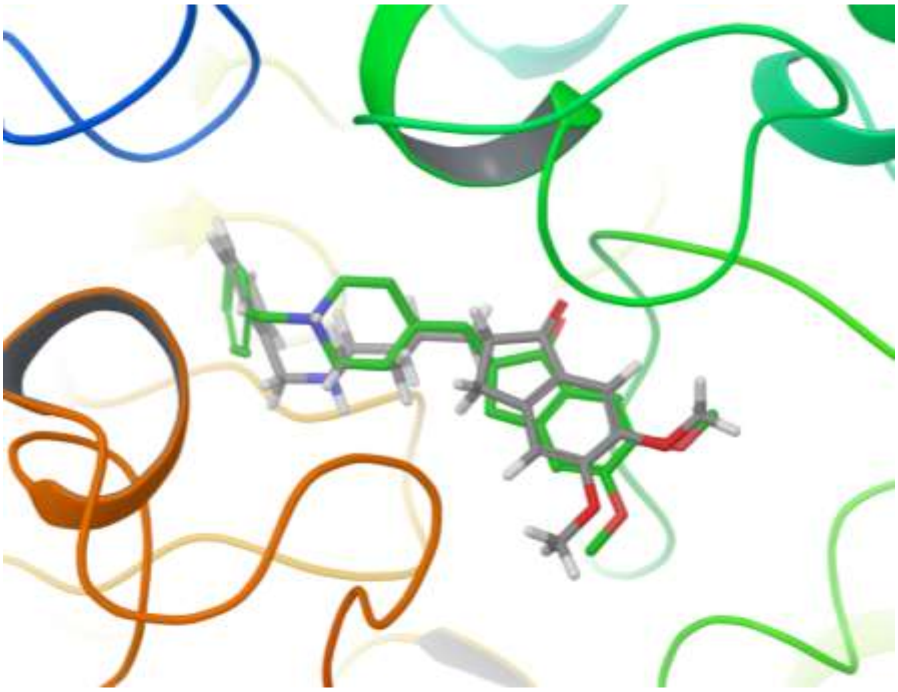

Figure S1. Superposition of the well-known inhibitor Donepezil, when it is docked in the *apo* state of AChE (PDB id: 4EY4) with the crystal deposited in the Protein Data Bank (PDB id: 4EY7).

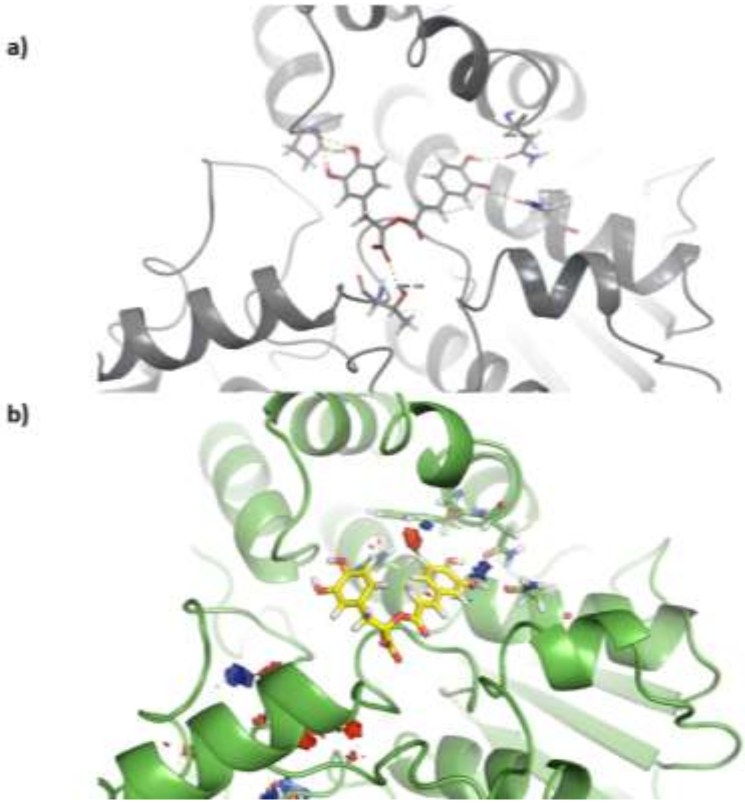

Figure S2. a) Pose for rosmarinic acid when glide docking is performed. **b)** Superimposition of the pose for rosmarinic acid with the hostspot calculated with Fragment Hotspots software. Donor hydrogen bond with Gln414 matches with the pose found.

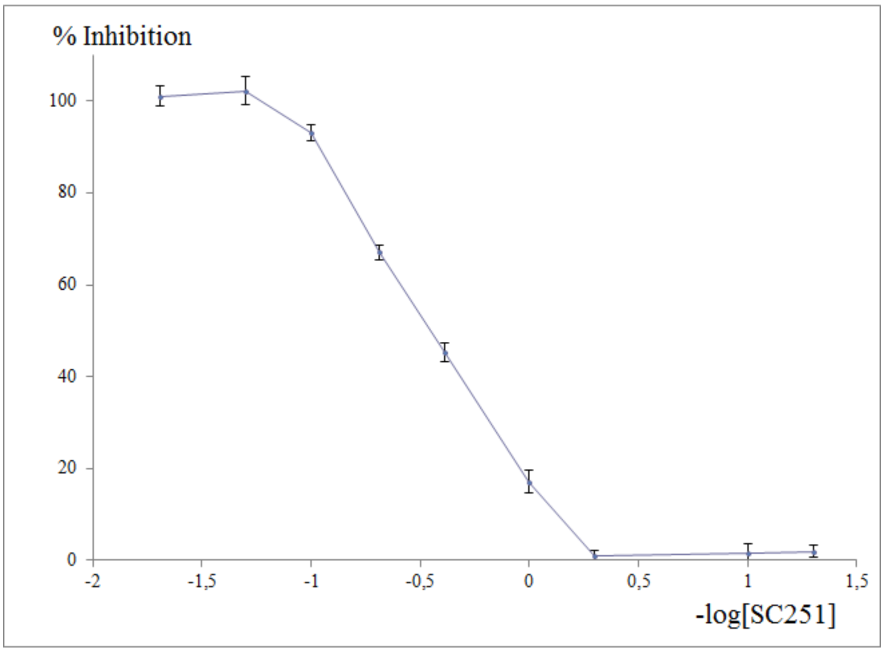

Figure S3. IC50 curve of the compound SC251. Inhibition value shows the residual activity of the enzyme on the vertical axis and the logarithm of the concentration on the horizontal axis.

Molecular dynamics simulations

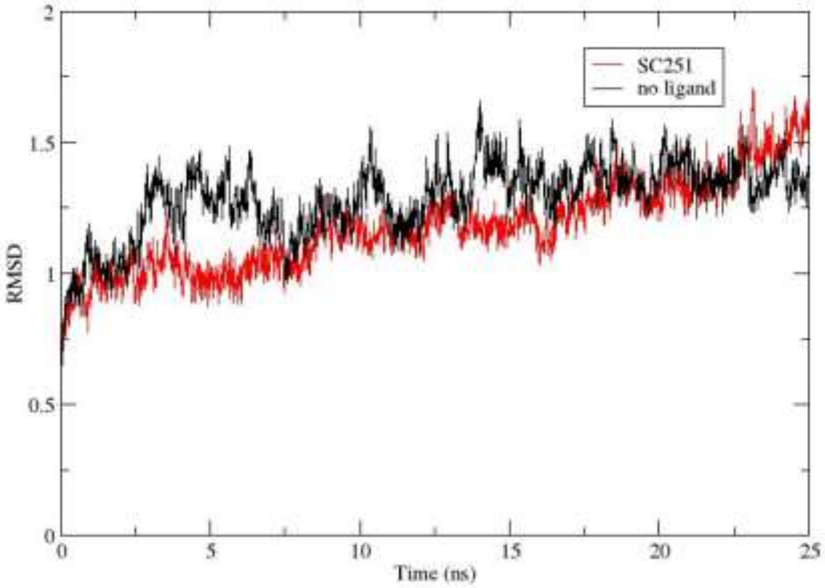

Figure S4. Cα RMSD data for the *apo* and ligand-protein complex hAChE-SC251 in MD simulations. After the initial adjustment, the longer-term values are stable.

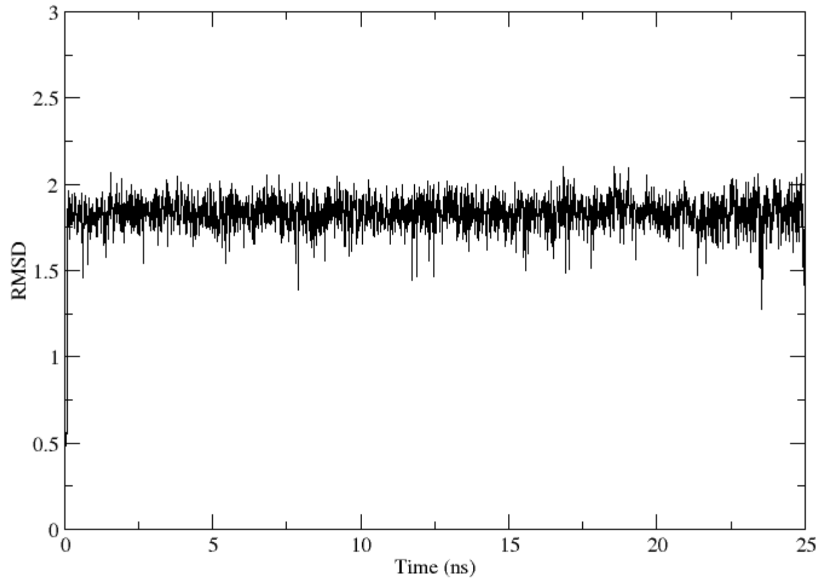

Figure S5. RMSD of SC251 along the MD simulation.

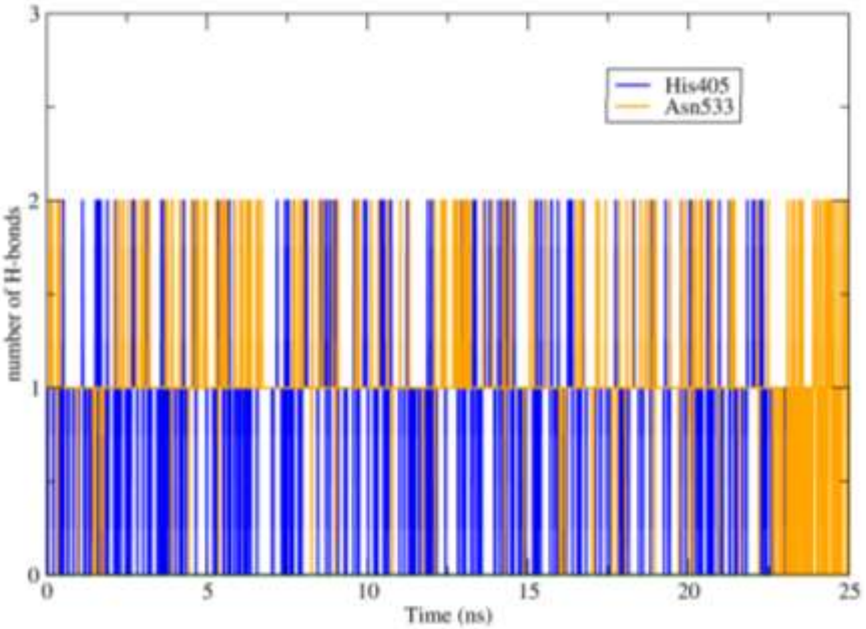

Figure S6. Number of hydrogen bonds between ligand SC251 and the residues His405 and Asn533 during the MD simulation. SC251 interacts with both residues, losing the ability to interact with Gln413.

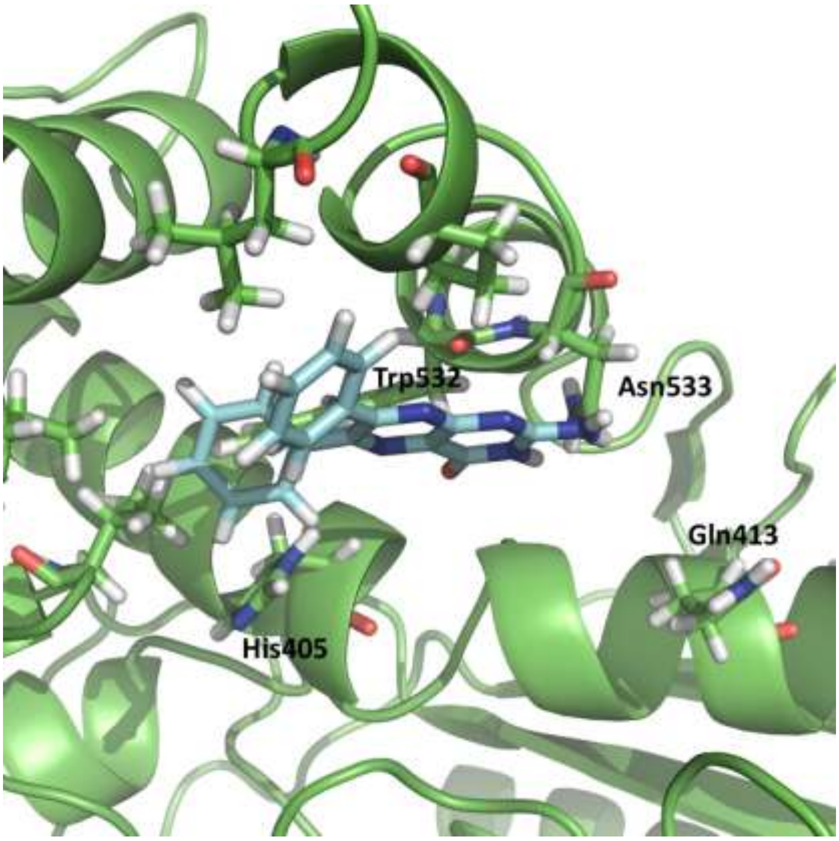

Figure S7. Stable ligand-protein complex between SC251 and hAChE found in MD simulation.

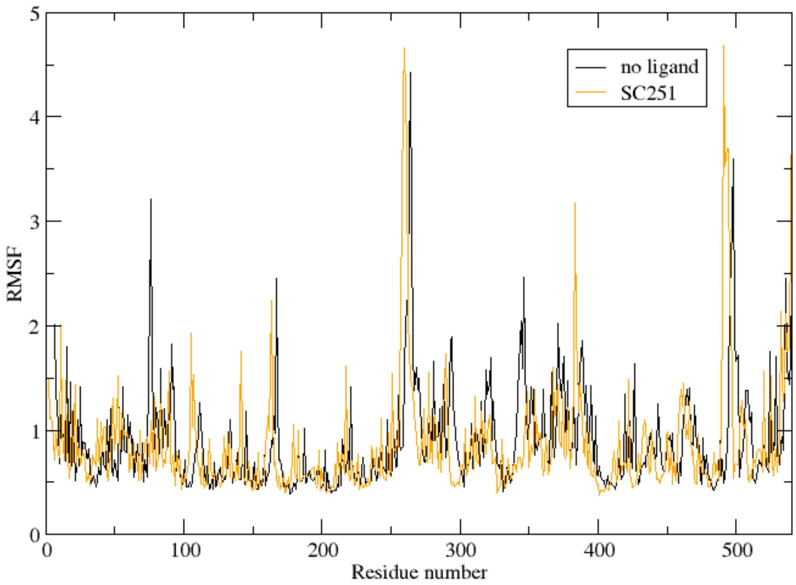

Figure S8. RMSF data for the *apo* and ligand-complex trajectories.

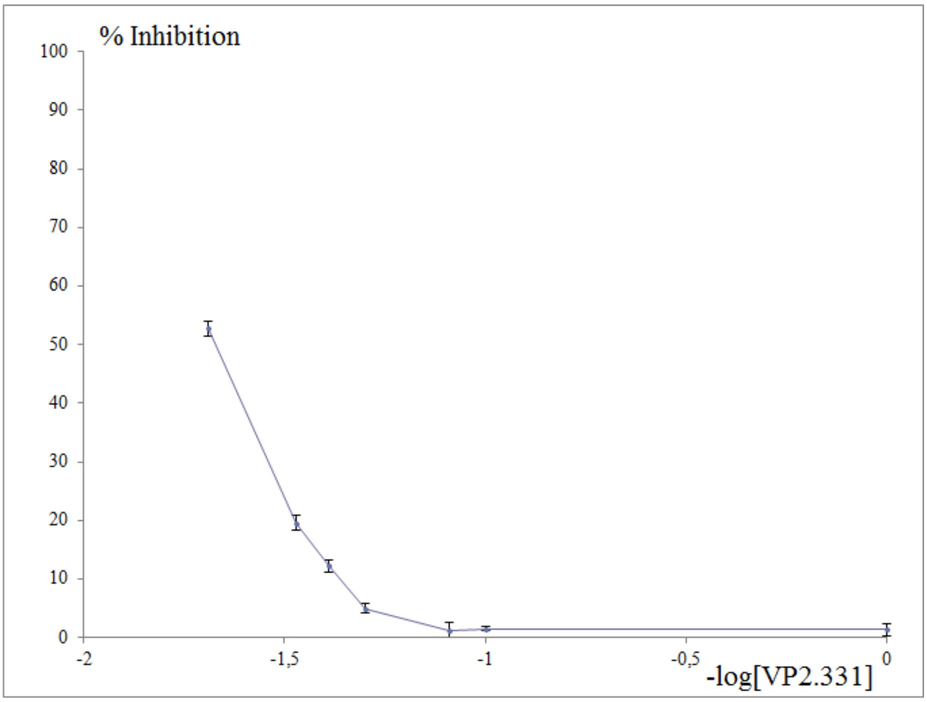

Figure S9. IC_{50} curve of the compound VP2.33. Inhibition value shows the residual activity of the enzyme on the vertical axis and the logarithm of the concentration on the horizontal axis.

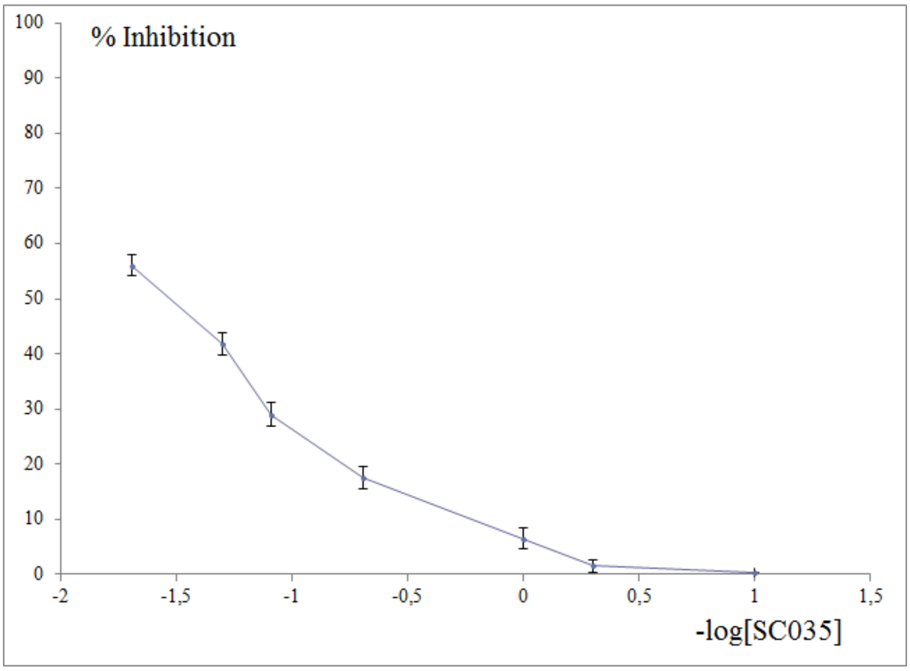

Figure S10. IC_{50} curve of the compound SC035. Inhibition value shows the residual activity of the enzyme on the vertical axis and the logarithm of the concentration on the horizontal axis.

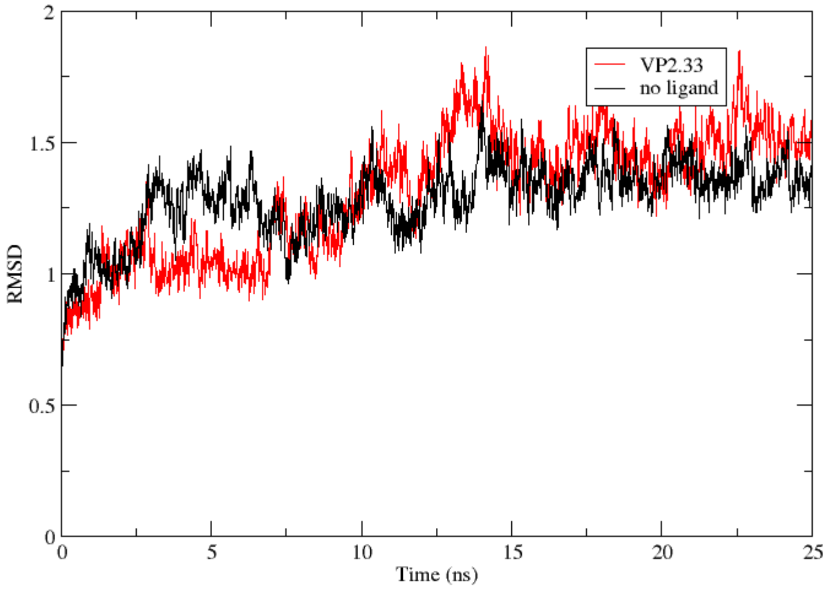

Figure S11. Cα RMSD data for the *apo* and ligand-protein complex hAChE-VP2.33 in MD simulations. After the initial adjustment, the longer-term values are stable.

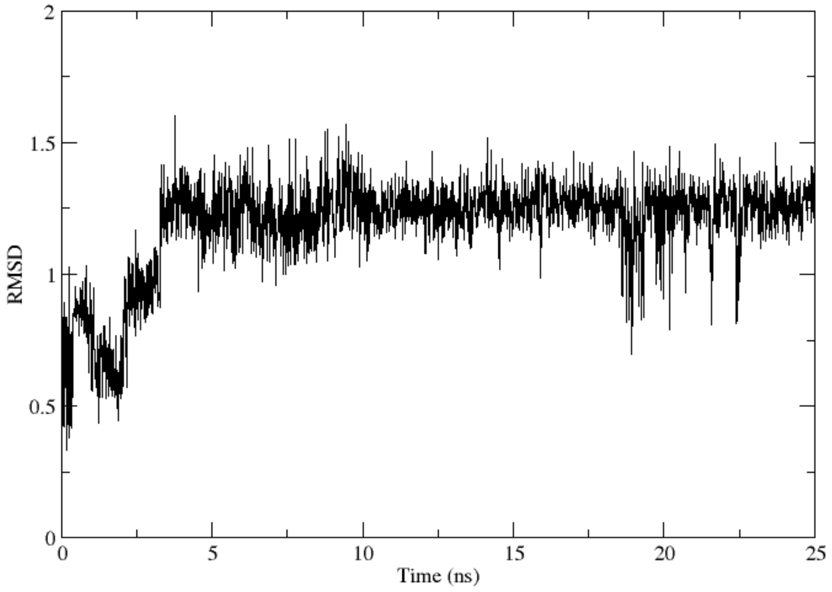

Figure S12. RMSD of VP2.33 along the MD simulation.

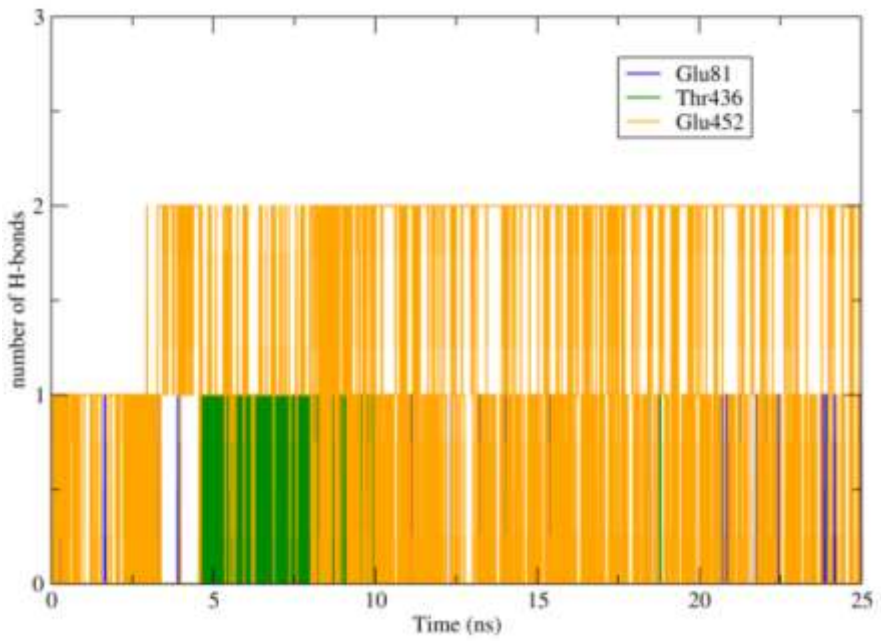

Figure S13. Number of hydrogen bonds between ligand VP2.33 and the residues Glu81, Thr436 and Glu452 during the MD simulation. VP2.33 interacts mainly with Glu452.

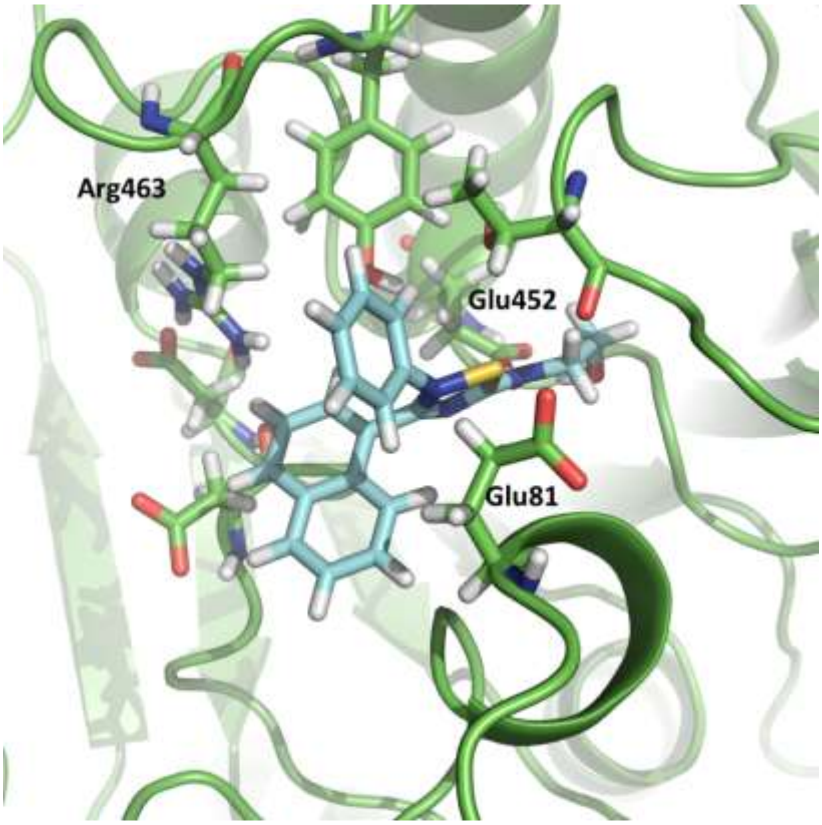

Figure S14. Stable ligand-protein complex between VP2.33 and hAChE found in MD simulation.

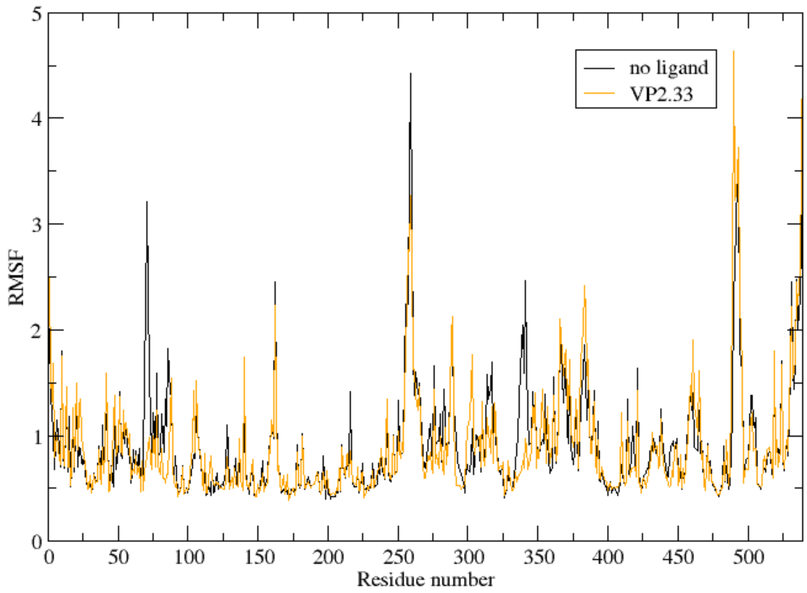

Figure S15. RMSF data for the *apo* and VP2.33-complex trajectories.

Table S1. Results of the inhibition of AChE with JTE907 and SC035. The value in brackets corresponds to individual inhibition of each compound.

	SC035 50 µM (58,72±1,24 %)	SC035 25 µM (42,33±3,27 %)
JTE907 20 µM (65,78±2,37 %)	62,44±2,75<br>ΔI_{JTE907}=-3,34<br>ΔI_{SC035}=3,72	62,32±1,65<br>ΔI_{JTE907}=-3,46<br>ΔI_{SC035}=19,99
JTE907 5 µM (34,22±1,13 %)	53,74±3,00<br>ΔI_{JTE907}=19,52<br>ΔI_{SC035}=-4,98	44,59±2,01<br>ΔI_{JTE907}=10,47<br>ΔI_{SC035}=2,26
JTE907 2,5µM (14,09±2,85 %)	47,09±1,50%<br>ΔI_{JTE907}=33<br>ΔI_{SC035}=-11,76	35,59±2,26<br>ΔI_{JTE907}=21,5<br>ΔI_{SC035}=-6,74
	JTE907 5µM (34,22±1,13 %)	JTE907 2,5µM (14,09±2,85 %)
SC035 70 µM (75,32±3,03 %)	54,55±2,19<br>ΔI_{SC035} =-20,77<br>ΔI_{JTE907}=20,33	62,11±1,09<br>ΔI_{SC035}=-13,21<br>ΔI_{JTE907}=48,02
SC035 50 µM (58,72±1,24 %)	51,42±3,75<br>ΔI_{SC035}= -7,3<br>ΔI_{JTE907}=17,2	48,00±2,07<br>ΔI_{SC035}= -10,72<br>ΔI_{JTE907}=33,91
SC035 25 µM (42,33±3,27 %)	(48,00±2,07 %)<br>ΔI_{SC035}= 5,67<br>ΔI_{JTE907}=13,78	32,50±2,84<br>ΔI_{SC035}= -6,83<br>ΔI_{JTE907}=18,41
